# The first complete mitochondrial genome of *Hexagenia rigida* Mc Dunnough, 1924 (Ephemeroptera: Ephemeridae) and its phylogeny

**DOI:** 10.1080/23802359.2022.2086498

**Published:** 2022-06-20

**Authors:** Yao Tong, Lian Wu, Yi-Jie Lin, Sam Pedro Galilee Ayivi, Kenneth B. Storey, Jia-Yong Zhang, Dan-Na Yu

**Affiliations:** a College of Chemistry and Life Science, Zhejiang Normal University, Jinhua, Zhejiang Province, China; b Department of Biology, Carleton University, Ottawa, Canada; c Key Lab of Wildlife Biotechnology, Conservation and Utilization of Zhejiang Province, Zhejiang Normal University, Jinhua, Zhejiang Province, China

**Keywords:** Ephemeridae, mitochondrial genome, phylogeny

## Abstract

The phylogenetic relationship of Ephemeridae (Insect: Ephemeroptera) remains hotly debated using mitochondrial (mt) genomes. All previously reported mt genomes of Ephemeridae belong to the genus *Ephemera*. This study provides the first complete mt genome sequence from the genus *Hexagenia* with an analysis of the mitogenome of *Hexagenia rigida* Mc Dunnough, 1924 (Ephemeroptera: Ephemeridae) and providing new information to discuss the phylogenetic relationships within Ephemeroptera. The complete mt genome of *H. rigida* was a circular molecule of 16,159 bp in length, containing 37 genes (2 rRNA genes, 13 protein-coding genes, 22 tRNA genes), which showed the typical mt gene arrangement of insects. The AT content of the whole genome was 70.0% and the length of the control region was 1091 bp. All protein-coding genes used ATN as the start codon, and most PCGs used TAA/TAG as the stop codons excluding COI, COII, ND5 and Cyt b that used T as the stop codon. BI and ML phylogenetic trees constructed from 27 species of 13 families showed that Ephemeridae is a sister clade to the clade Polymitarcyidae.

Ephemeroptera has a worldwide distribution, occurring on all continents except Antarctica (Ratnasingham & Hebert 2007). As a relatively primitive group of Pterygota, the phylogenetic relationship of Ephemeroptera has always been a research hotspot (Hebert et al. 2003; Ogden and Whiting 2005; Sun et al. 2006; O’Donnell and Jockusch 2008; Ogden et al. 2009; Webb et al. 2012; Saito et al. 2016; Cai et al. 2018; Gao et al. 2018; Ye et al. 2018; Xu et al. 2019; Guan et al. 2021; Xu et al. 2021; Yu et al. 2021). Considerable effort has been devoted to constructing the phylogenetic relationships among Ephemeroptera families based on morphology (Mccafferty 1991; Mccafferty and Edmunds 1979), molecular evidence (Ogden and Whiting 2005), and combined data (Ogden et al. 2009; Xu et al. 2020). However, there are relatively few studies of the phylogenetic relationships within the Ephemeridae. To date, no mitochondrial (mt) genome of genus *Hexagenia* has been reported. In this study, we sequenced the first mt genome from this genus (*Hexagenia rigida* Mc Dunnough, 1924) and discuss its phylogenetic relationship within Ephemeridae.

The female imago of *H. rigida* (Sample Number: WTH201707) was collected by JY Zhang using sweeping net at Carleton University, Ottawa (45°38′ N 75°69′ W), Canada on 15 July 2017. Insects used in this study are not regulated. The sample was identified and stored at −40 °C freezer in the Animal Specimen Museum, College of Life Sciences and Chemistry, Zhejiang Normal University, China. Total genomic DNA was extracted from individual tissues of the sample using an Ezup Column Animal Genomic DNA Purification Kit (Sangon Biotech Company, Shanghai, China) and stored in the Zhang laboratory (http://mypage.zjnu.edu.cn/ZJY3/zh_CN/index.htm, Zhang JY, zhang3599533@163.com). Universal primers were used to amplify some partial fragments as described in Zhang et al. (2008). Subsequently, the remaining gaps were sequenced by utilizing specific primers according to previously obtained sequences. Manual proofreading and splicing of all nucleotide fragments were conducted using SeqMan in the DNASTAR Package (Burland 2000). Annotation of all mitochondrial genes were identified by the online website MITOS (http://mitos.bioinf.uni-leipzig.de/index.py) (Bernt et al. 2013). The mt genome was deposited in GenBank with accession number OL678102.

The complete mt genome of *H. rigida* was 16,159 bp in length, which is similar to all known mt genomes of Ephemeridae. It encoded of 37 genes including 13 protein-coding genes (PCGs), 22 transfer RNAs (tRNAs), two ribosomal RNAs (rRNAs), and one control region (CR). The whole mt genome and the control region of *H. rigida* had a high AT content of 70.0% and 68.7%, respectively. The total length of the PCGs was 11,199 bp and all genes showed a negative AT-skew. Nine of the PCGs (ND2, COI, COII, ATP8, ATP6, COIII, ND3, ND6, and Cyt b) were located on the heavy strand (H-strand), whereas the others (ND5, ND4, ND4L, and ND1) were located on the light strand (L-strand). The start codons of the PCGs in *H. rigida* were ATG (in COII, COIII, ND5, ND4, ND4L, Cyt b, and ND1), ATT (in ATP8, ND3, and ND6), and ATA (in ND2 and ATP6). The typical stop codons (TAA and TAG) were used in nine PCGs. However, an incomplete stop codon T occurred in four genes (COI, COII, ND5, and Cyt b). It is quite common in insect mt genomes to use an incomplete stop codon. These truncated stop codons are presumed to be completed by post-transcriptional polyadenylation (Ojala et al. 1981). The summed lengths of the 22 tRNAs, two rRNAs and the CR were 1456 bp, 2131 bp, and 1090 bp, respectively. The 16S RNA and 12S RNA genes were located between tRNA-Leu and tRNA-Val, and between tRNA-Val and the CR, respectively. In the whole mt genome, we found nine overlapping areas each ranging from 1 to 8 bp and the gene arrangement was identical to the ancestral insect gene pattern.

The phylogenetic relationship was constructed by Bayesian inference (BI) using MrBayes 3.1.0 (Huelsenbeck and Ronquist 2001) and maximum-likelihood (ML) using RAxML 8.2.0 (Stamatakis 2014) based on the 13 PCGs. Twenty-seven mt genomes within Ephemeroptera were downloaded from GenBank (Li et al. 2014; Tang et al. 2014; Ye et al. 2018; Cao et al. 2020; Li et al. 2020; Macher et al. 2020; Xu et al. 2020; Yu et al. 2021; Tong et al. 2022) and were used to investigate the phylogenetic relationships. In addition, *Siphluriscus chinensis* (HQ875717 and MF352165), the most primitive family of the Ephemeroptera, was used as the outgroup. Each alignment was performed using Gblock 0.91b (Castresana 2000) with default settings. The phylogenetic relationship based on BI and ML analyses (Figure 1) indicated that almost all families were monophyletic, including Ephemeridae. According to the results of phylogenetic topologies, Isonychiidae was the basal clade to Ephemeroptera excluding the outgroup Siphluriscidae. After that, Ameletidae and Siphlonuridae were found to be a sister group. Heptageniidae as a sister clade to the remaining Ephemeroptera (Heptageniidae + (((Leptophlebiidae + (Caenidae + (Teloganodidae + Baetidae))) + (Ephemerellidae + Vietnamellidae)) + (Potamanthidae + (Polymitarcyidae + Ephemeridae))). Analysis of phylogenetic revealed that Ephemeridae was shown to be a sister clade to the clade of Polymitarcyidae and *H. rigida* was a sister clade to genus *Ephmera*.

**Figure 1.**
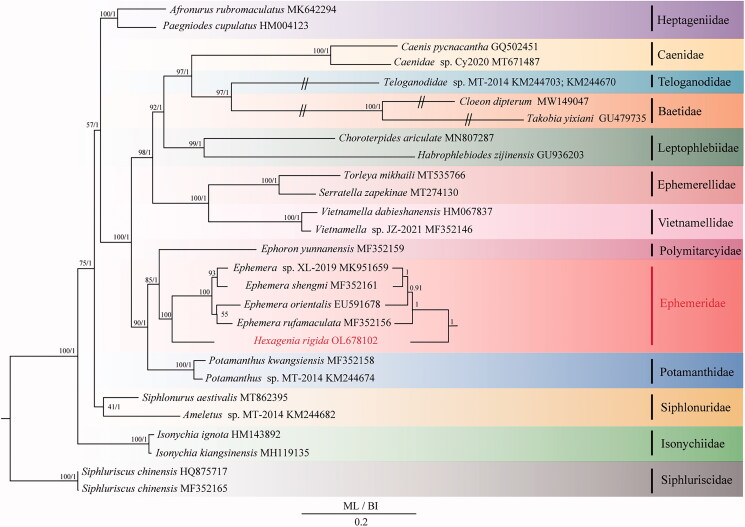
The phylogenetic relationships of BI and ML trees using 28 species of Ephemeroptera, including *H. rigida* (OL678102) and based on the nucleotide dataset of the 13 mt PCGs. *Siphluriscus chinensis* (HQ875717 and MF352165) was used as the outgroup. The numbers above branches specify posterior probabilities as determined from BI (left) and bootstrap percentages from ML (right). The GenBank accession numbers of all species are shown in the figure. The long-branch attractions of Baetidae and Teloganodidae have been cut for esthetics.

## Data Availability

The mitochondrial genome data that support the findings of this study are openly available in GenBank of NCBI at [https://www.ncbi.nlm.nih.gov/nuccore/OL678102] under the accession no. OL678102. The mt genome was obtained by the Sanger method, so no associated “BioProject,” “SRA,” and “Bio-Sample” numbers should be shown.
